# The coronavirus pandemic in Israel: A comparison between holocaust survivors and other older adults

**DOI:** 10.1192/j.eurpsy.2021.294

**Published:** 2021-08-13

**Authors:** E. Cohn-Schwartz, Y. Bachner, S. Carmel

**Affiliations:** 1 Public Health, Ben-Gurion University, Beer-Sheva, Israel; 2 Multidisciplinary Research Center On Aging, Ben-Gurion University, Beer-Sheva, Israel

**Keywords:** COVID-19, Holocaust survivors, mental health, loneliness

## Abstract

**Introduction:**

The COVID-19 pandemic places older adults at increased risk for hospitalization and mortality. It also involves social isolation and negative effects of limited mental, social and physical activity. Holocaust survivors could be especially vulnerable to such effects due to their early life traumas. Previous research suggests that in times of life crises, Holocaust survivors may be both most vulnerable (i.e., wear-and-tear hypothesis); yet they may also demonstrate resilience.

**Objectives:**

Thus, the current study examines the effects of the COVID-19 pandemic on the mental health and well-being of Holocaust survivors in Israel, compared to adults who did not experience the Holocaust.

**Methods:**

We collected data from 305 older adults aged 75 and above in Israel during the COVID-19 pandemic. Of these, 114 were Holocaust survivors and 191 did not experience the Holocaust. Participants were asked about their worries of COVID-19 infections, will to live, loneliness and depression and how these changed during the COVID-19 pandemic.

**Results:**

Holocaust survivors were worried to a greater extent from COVID-19 infection and from close others becoming infected, compared to older adults who did not experience the Holocaust. Moreover, survivors reported greater loneliness and depression overall and also reported that these measures became worse during the pandemic. On the other hand, despite these differences, the two groups were similar in their will to live.

**Conclusions:**

Holocaust survivors seem to be more vulnerable to the COVID-19 pandemic, strengthening the vulnerability hypothesis. Policy makers and practitioners should pay special attention to this particularly vulnerable population during these difficult times.

**Disclosure:**

No significant relationships.

